# Conjoint moderate or high-risk alcohol and tobacco use among male out-patients in Thailand

**DOI:** 10.4102/sajpsychiatry.v22i1.763

**Published:** 2016-03-22

**Authors:** Supa Pengpid, Karl Peltzer, Apa Puckpinyo, Kriengsak Thammaaphiphol

**Affiliations:** 1 ASEAN Institute for Health Development, Mahidol University, Salaya, Phutthamonthon, Nakhonpathom, Thailand; 2 Department of Research Innovation and Development, University of Limpopo, Turfloop campus, South Africa; 3 Department of Psychology, University of Limpopo, Turfloop Campus, South Africa; 4 HIV/AIDS/STIs and TB (HAST), Human Sciences Research Council, South Africa

## Abstract

**Objective:**

To better understand conjoint alcohol and tobacco use among male hospital out-patients, the purposes of this study were: (1) to assess the prevalence of conjoint use and (2) to determine the factors associated with the conjoint alcohol use and tobacco use.

**Methods:**

In a cross-sectional survey, consecutive male out-patients from four district hospitals in Nakhon Pathom province in Thailand were assessed with the Alcohol, Smoking and Substance Involvement Screening Test (ASSIST), Hospital Anxiety and Depression Scale (HADS), self-reported chronic conditions and health-seeking behaviour. The sample included 2208 study participants, with a mean age of 36.2 years (SD = 11.7) and an age range of 18–60 years.

**Results:**

Overall, 34.5% of the male hospital out-patients were conjoint moderate or high-risk alcohol and tobacco users, and 31.1% were moderate or high-risk alcohol or tobacco users. In multivariate analysis, younger age, having primary or less education, being separated, divorced or widowed, not having diabetes and not being obese were associated with conjoint moderate or high-risk alcohol and tobacco use.

**Conclusion:**

High prevalence and several risk factors of conjoint alcohol and tobacco use were found among hospital male out-patients. The findings of this study call for dual-intervention approaches for both alcohol and tobacco.

## Introduction

In Thailand the age-standardised prevalence of joint (daily) smoking and harmful or hazardous alcohol consumption in adults over 14 years of age was among men 10.0% and 10.3% in urban and rural areas, respectively, and among women 0.2% and 0.3% in urban and rural areas, respectively.^1^ In primary care patients in Thailand the most popular substances were tobacco and alcohol;^2^ in southern Thailand 90.5% were moderate or high-risk tobacco users and 44.6% were moderate or high-risk alcohol users.^3^ Among general hospital patients in Brazil the rate of comorbidity between alcohol use disorder and nicotine dependence was 3.6%;^4^ in primary health care TB patients in South Africa it was 15.5%,^5^ and the prevalence of concurrent alcohol and tobacco use in urban areas was, among men, 20.1% and in rural areas 14% in Sri Lanka.^6^ Previous studies^7^ have found that the synergistic health risks associated with alcohol and tobacco use are estimated to be 50% higher than the sum of their independent risks. There is a high association between nicotine and alcohol dependence.^8,9,10^ Compared to one specific substance use dependence, conjoint nicotine and alcohol dependence is more severe and has a more unfavourable course.^9^ Hurley et al.’s^11^ review showed that abuse of alcohol and nicotine (from tobacco products) can be attributed in part to genetics, the reward system and possibly the analgesic effects the drugs have.

Conjoint alcohol and tobacco use has been found to be associated with lower education,^12,13^ being male,^12,13^ younger age,^14,15^ and mental problems, including depression and anxiety disorders.^12,13,16,17,18^ Further, in general, people with a substance use disorder also have higher comorbid rates of mental disorders than vice versa.^8,10,19^ There is a lack of studies investigating conjoint alcohol and tobacco use among hospital male out-patients. Therefore, the aim of this study was exploring the prevalence of conjoint alcohol and tobacco use and alcohol or tobacco use and to determine the factors associated with conjoint alcohol and tobacco use and alcohol or tobacco use among male out-patients in Thailand.

## Method

### Sample and procedure

#### Ethical considerations

Four out of eight district hospitals in the Nakhon Pathom province in Thailand were randomly selected. In a cross-sectional survey consecutive male clients visiting the district hospital out-patient department were included in the study. A health care provider informed the patient about the study and referred the patient for participation if interested. A research assistant asked for permission and consent from patients attending the district hospital facility to participate in the study. Sampling occurred throughout all hours of hospital out-patient operation over a four-month period. We received ethical approval from the Mahidol University Research and Ethics Committee (COA. No. 2014/111.1804). The study was conducted from May 2014 to August 2014.

#### Measures

*Tobacco and alcohol use* were assessed with the Alcohol, Smoking and Substance Involvement Screening Test (ASSIST).^20^ A risk score is determined for each substance and is categorised as low-risk, moderate-risk or high-risk.^20^ In addition to tobacco and alcohol, other substances, according to the ASSIST, were also assessed.^20^ Further, patients were asked about their health care seeking behaviour for alcohol or tobacco use problems in the past three months. Those who were using alcohol and tobacco were asked about their needs to smoke in the context of drinking alcohol.

*Socioeconomic characteristics:* Age, gender, educational level, marital status, income, employment status and religious affiliation were assessed.

*Chronic conditions:* Major chronic conditions diagnosed by a health care provider, for example diabetes or hypertension, were assessed by self-report.

The *Hospital Anxiety and Depression Scale* (HADS)^21^ was used to assess anxiety (seven items) and depression (seven items). Scores of 8 or more were used as cut-offs for anxiety and depression.^22^ The HADS has good reliability and validity in Thailand.^22,23^ HADS subscales were internally reliable (α depression: 0.74; α anxiety: 0.77).

### Data analysis

The data were analysed using IBM SPSS (version 20.0)^24^. Frequencies, means and standard deviations were calculated to describe the sample. Associations of conjoint moderate or high-risk alcohol use and tobacco use, and moderate or high-risk alcohol use or moderate or high-risk tobacco use (without dual moderate or high-risk alcohol and tobacco use) were identified using logistic regression analysis. Following each univariate regression, multivariable regression models were constructed. Independent variables from the univariate analysis were entered into the multivariable model if significant at a level of P less than 0.05. For each model, the R^2^ values are presented to describe the amount of variance explained by the multivariable model. Probability below 0.05 was regarded as statistically significant.

## Results

Overall, 34.5% of the male hospital out-patients were conjoint moderate or high-risk alcohol and tobacco users, and 31.1% were moderate or high-risk alcohol or tobacco users (see Table 1). The use of other substances, as assessed with the ASSIST, found lifetime and past three months use as follows: 9.1% and 0.2% for cannabis, 0.7% and 0% for cocaine, 6.9% and 0.1% for amphetamine, 1.5% and 0% for solvents, 0.7% and 0.1% for diazepam or sleeping pills, 0.2% and 0% for hallucinogens, 0.8% and 0% for opium and 1.3% and 0.1% for other addictive substances. Other conditions that had been diagnosed included chronic obstructive pulmonary disease (COPD) (0.5%), coronary artery disease (1.0%), cardiac failure (0.3%), cardiac arrhythmias (0.6%), stroke (0.5%), arthritis (1.2%), cancer (0.2%), thyroid disease (0.7%), liver disease (1.9%), kidney disease (0.4%), epilepsy (0.4%), stomach and intestinal disease (4.4%), migraine or frequent headaches (3.8%), and mental disorder (0.7%).

**TABLE 1:** Sample characteristics, moderate and high-risk alcohol or tobacco use by independent variables in male out-patients, 2014.

Sociodemographic variables	Total *N* (%)	Moderate or high-risk alcohol and tobacco user *N* (%)	Moderate or high-risk alcohol or tobacco user *N* (%)
**All**	2208	765 (34.5)	686 (31.1)
**Age in years**			
18–25	522 (23.6)	219 (42.0)	158 (30.3)
26–34	527 (23.9)	199 (37.8)	171 (32.4)
35–44	567 (25.7)	193 (34.0)	182 (32.1)
45–60	592 (26.8)	151 (25.5)	175 (29.6)
**Education**			
Primary or less	1157 (52.4)	410 (35.4)	356 (30.8)
Secondary	849 (38.5)	307 (36.2)	276 (32.5)
Postsecondary	202 (9.1)	45 (22.3)	54 (26.7)
**Marital status**			
Never married	571 (25.9)	193 (33.8)	169 (29.6)
Married or cohabiting	1569 (71.1)	536 (34.2)	500 (31.9)
Separated/Divorced/Widowed	68 (3.1)	33 (48.5)	17 (25.0)
**Religious affiliation**: Buddhist (vs. others)	2187 (99.1)	754 (34.5)	681 (31.1)
**Employed (vs. unemployed or other)**	2038 (92.5)	713 (35.0)	634 (31.1)
**Monthly income**			
< 10 000 Baht	610 (27.7)	211 (34.6)	209 (34.3)
10 000–15 000 Baht	796 (36.1)	304 (38.2)	226 (28.4)
> 15 000 Baht	797 (36.2)	245 (30.7)	249 (31.2)
**Chronic conditions**			
Diabetes	133 (6.0)	22 (16.5)	35 (26.3)
Hypertension	286 (13.0)	71 (24.8)	85 (29.7)
Dyslipidemia	191 (8.7)	46 (24.1)	58 (30.4)
Asthma	107 (4.9)	44 (41.1)	31 (29.0)
Gout and other musculoskeletal conditions, such as chronic backache	131 (5.9)	46 (35.1)	50 (38.2)
**Mental problems**			
Anxiety	107 (4.8)	46 (43.0)	36 (33.6)
Depression	57 (2.6)	19 (33.3)	25 (43.9)

*Source*: Author’s own work.

The proportion of moderate or high-risk alcohol users was 44.2%, and the proportion of moderate or high-risk tobacco users was 55.8%. The proportion of moderate or high-risk tobacco users among moderate or high-risk alcohol users was 78.0%, and 61.8% were moderate or high-risk alcohol users among the moderate or high-risk tobacco users. Compared to the older age group (35–60 years), the younger age group (18–34 years) was significantly more likely to engage in moderate or high-risk alcohol or tobacco use (see Table 2). The ASSIST alcohol use means score among conjoint moderate or high-risk alcohol and tobacco users was higher (M = 17.0, SD = 4.3) than among moderate or high-risk alcohol users who were not moderate or high-risk tobacco users (M = 16.8, SD = 4.3). Likewise, the ASSIST tobacco use means score among conjoint moderate or high risk alcohol and tobacco users was higher (M = 20.1, SD = 5.4) than among moderate or high-risk tobacco users who were not moderate or high-risk alcohol users. Those with moderate or high-risk alcohol use had a significantly higher odds of anxiety symptoms (odds ratio (OR) 1.58; confidence interval (CI) 1.07–2.34). From the total sample of male outpatients, 2.5% reported to have consulted the self-help or quit line for their tobacco use problem, 1.2% a health facility and 0.3% had consulted a traditional or complementary medicine provider in the past three months. Regarding their alcohol problems, nobody had consulted a health care provider and 0.2% had consulted a traditional or complementary medicine provider in the past three months. The tendency or urge to smoke when drinking alcohol was felt by 38.3% of the study participants every time, 27.1% most of the time, 6% half of the time, 10.3% sometimes, and 18.3% never.

**TABLE 2:** Alcohol and tobacco user overlap.

Variable	Total sample		Age: 18–34		Age: 35–60		Statistic	
			
	*N*	%	*N*	%	*N*	%	*P*-value
Moderate or high-risk alcohol users	977	44.2	513	48.9	464	40.0	< 0.001
Moderate or high-risk tobacco users	1233	55.8	652	62.2	581	50.1	< 0.001
Proportion of moderate or high-risk tobacco users among moderate or high-risk alcohol users	762	78.0	418	81.5	344	74.1	0.006
Proportion of moderate or high-risk alcohol users among moderate or high-risk tobacco users	762	61.8	418	64.1	344	59.2	0.077
Moderate or high-risk alcohol users or moderate or high-risk tobacco users	686	31.1	329	31.4	357	30.8	0.776
Moderate or high-risk alcohol users and moderate or high-risk tobacco users	762	34.5	418	39.8	344	29.7	< 0.001

*Source*: Author’s own work.

### Associations with moderate or high-risk alcohol or tobacco use

In multivariate analysis in terms of socio-demographics, younger age (OR = 0.69; CI = 0.53–0.90), having primary or less education (OR = 0.46; CI = 0.32–0.66) and being separated, divorced or widowed (OR = 2.61; CI = 1.53–4.44) were found to be associated with conjoint moderate or high-risk alcohol and tobacco use, while lower monthly income (OR = 0.76; CI = 0.61–0.95) was associated with moderate or high-risk alcohol or tobacco use. In relation to chronic conditions, not having diabetes (OR = 0.51; CI = 0.31–0.83) was in the multivariate analysis associated with conjoint moderate or high-risk alcohol and tobacco use. Finally, in terms of mental problems, depressive symptoms (OR = 1.76; CI = 1.03–2.99) were associated with moderate or high-risk alcohol or tobacco use (see Table 3).

**TABLE 3:** Association of sociodemographic and health variables and moderate or high-risk alcohol or tobacco use among male out-patients, 2014 (*N* = 2180).

Sociodemographic variables	Moderate or high-risk alcohol and tobacco user		Moderate or high-risk alcohol or tobacco user	
	
	UOR (95% CI)	AOR (95% CI)^a^	UOR (95% CI)	AOR (95% CI)^b^
**Age in years**				
18–25	1.00	1.00	1.00	-
26–34	0.84 (0.66–1.08)	0.82 (0.63–1.06)	1.11 (0.85–1.44)	
35–44	0.71 (0.56–0.91)**	0.69 (0.53–0.90)**	1.09 (0.84–1.41)	
45–60	0.47 (0.37–0.61***	0.45 (0.33–0.61)***	0.97 (0.75–1.25)	
**Education**				
Primary or less	1.00	1.00	1.00	-
Secondary	1.03 (0.86–1.24)	0.86 (0.71–1.05)	1.08 (0.90–1.31)	
Post-secondary	0.52 (0.37–0.74)***	0.46 (0.32–0.66)***	0.82 (0.59–1.15)	
**Marital status**				
Never married	1.00	1.00	1.00	
Married or cohabiting	1.02 (0.83–1.04)	1.25 (0.99–1.58)	1.11 (0.90–1.37)	
Separated/Divorced/Widowed	1.85 (1.11–3.06)*	2.61 (1.53–4.44)**	0.79 (0.45–1.41)	
**Religious affiliation**: Buddhist (vs. others)	0.79 (0.32–1.94)	-	1.36 (0.49–3.75)	-
**Employed (vs. unemployed or other)**	1.31 (0.93–1.86)	-	1.07 (0.76–1.51)	-
**Monthly income**				
< 10 000 Baht	1.00	-	1.00	1.00
10 000–15 000 Baht	1.17 (0.94–1.46)		0.76 (0.61–0.96)*	0.76 (0.61–0.95)*
> 15 000 Baht	0.84 (0.67–1.05)		0.87 (0.70–1.09)	0.87 (0.69–1.09)
***Chronic conditions***
Diabetes	0.36 (0.22–0.57)***	0.51 (0.31–0.83)**	0.78 (0.53–1.16)	-
Hypertension	0.59 (0.44–0.78)***	0.91 (0.66–1.25)	0.93 (0.71–1.22)	-
Dyslipidemia	0.58 (0.41–0.81)	-	0.97 (0.70–1.33)	-
Asthma	1.34 (0.90–1.90)	-	0.90 (0.59–1.38)	-
Gout and other musculoskeletal conditions, such as chronic backache	1.03 (0.71–1.49)	-	1.40 (0.97–2.01)	-
**Mental problems**				
Anxiety	1.46 (0.98–2.16)	-	1.13 (0.75–1.71)	-
Depression	0.95 (0.54–1.65)	-	1.76 (1.04–2.99)*	1.76 (1.03–2.99)*

*Source*: Author’s own work.

UOR = unadjusted odds ratio; AOR = adjusted odds ratio; CI = confidence interval.

*** *P* < 0.001;

** *P* < 0.01;

* *P* < 0.05

a Hosmer and Lemeshow chi-square = 5.22, *p* = 0.733; Nagelkerke *R*^2^ = 0.06.

b Hosmer and Lemeshow chi-square = 9.19, *p* = 0.327; Nagelkerke *R*^2^ = 0.07.

## Discussion

This study found a high prevalence of conjoint alcohol and tobacco use in a large sample of male hospital out-patients in Thailand. Lower rates of comorbidity between alcohol use disorder and nicotine dependence were found among general hospital patients in Brazil,^4^ in primary health care TB patients in South Africa,^5^ and among men in the general population in Thailand^1^ and Sri Lanka.^6^ Similarly high rates of moderate or high-risk tobacco or alcohol use were found in a study among primary care attendees in southern Thailand.^3^ The study found that hardly any of the male out-patients had been seeking any type of health care service for their alcohol and tobacco use problem. This finding calls for screening and brief interventions,^2^ and providing dual-intervention approaches for both alcohol and tobacco.

Further, the study found, in agreement with other studies,^9^ that conjoint alcohol and tobacco users had higher ASSIST alcohol use and tobacco use scores than individuals who were tobacco or alcohol users only. Most participants in the study (65.4%) had the urge to smoke when drinking all the time or most of the time. Simultaneous administration of alcohol and nicotine may cause additive or synergistic analgesic effects.^11,25^ Another contributory factor to co-use of alcohol and nicotine is likely the additive or synergistic activation of the reward system.^11^ This could mean that more intensive intervention strategies are needed, possibly integrating both alcohol reduction and tobacco cessation.

Several risk factors (socio-demographic and health factors, but not mental problems) were jointly associated with alcohol and tobacco use. In agreement with other studies,^12,13,14,15^ this study also found that lower education and younger age were associated with conjoint alcohol and tobacco use and lower monthly income was associated with moderate or high-risk alcohol or tobacco use. The finding that being separated, divorced or widowed was found to be associated with conjoint moderate or high-risk alcohol and tobacco use could be explained by substance use being a coping mechanism for their detrimental family situation. In relation to chronic conditions, not having diabetes was in the multivariate analysis associated with conjoint moderate or high-risk alcohol and tobacco use. It is plausible that male out-patients who have been diagnosed with diabetes may have reduced or stopped tobacco or alcohol use.

Unlike in some previous studies,^12,13,16,17,18^ this study did not find any association between mental problems (anxiety and depression) and joint alcohol and tobacco use. However, there was an association between depression and moderate or high-risk alcohol or tobacco use, and those with moderate or high-risk alcohol use had significantly higher odds of anxiety symptoms.

### Study limitations

This study had several limitations. The study was cross-sectional, so causal conclusions cannot be drawn. The investigation was carried out with male out-patients from four hospitals in one province, and inclusion of other hospitals could have resulted in different results. Hospital male out-patients are not representative of male adults in general, and the prevalence of the different alcohol and tobacco use practices and their risk factors may be different in other sectors of the population. Male patients consulting a hospital out-patient department with various physical illnesses may have a greater likelihood of smoking and alcohol drinking and this may thus be over-represented in this sample. Another limitation of the study was that all the other information collected in the study was based on self-reporting. Self-reported data may be unreliable and thereby limit the validity of the associations drawn between conjoint substance use and other physical ailments.

### Conclusion

High prevalence and several risk factors of conjoint alcohol and tobacco use were found among male hospital out-patients. The hospital out-patient setting may be a good place to carry out adequate dual-intervention approaches for both alcohol and tobacco use in men.
